# Supplementary material for: Mechanical response of dense pedestrian crowds to the crossing of intruders
Source: Sci Rep. 2019 Jan 14;9:105. doi: 10.1038/s41598-018-36711-7 (PMC6331639; doi:10.1038/s41598-018-36711-7)
Supplement: Supplementary file 1 — Supplementary Information [file 41598_2018_36711_MOESM1_ESM.pdf]

Supplementary Information for: Mechanical response of dense pedestrian crowds to the crossing of intruders

Alexandre NICOLAS[1,*], Marcelo KUPERMAN[2], Santiago IBAÑEZ[2,3], Sebastián BOUZAT[2], and Cécile APPERT-ROLLAND[4]

[1]LPTMS, CNRS, Université Paris-Sud, Université Paris-Saclay, 91405 Orsay, France.
[2]Consejo Nacional de Investigaciones Científicas y Técnicas, Centro Atómico Bariloche (CNEA) and Instituto Balseiro, R8400AGP Bariloche, Argentina.
[3]Universidad Nacional de Río Negro, Sede Andina, 8400 Bariloche, Argentina.
[4]LPT, CNRS UMR 8627, Université Paris-Sud, Université Paris-Saclay, 91405 Orsay, France.
[*]alexandre.nicolas@polytechnique.edu

ABSTRACT

We provide three additional figures as Supplemental Material.

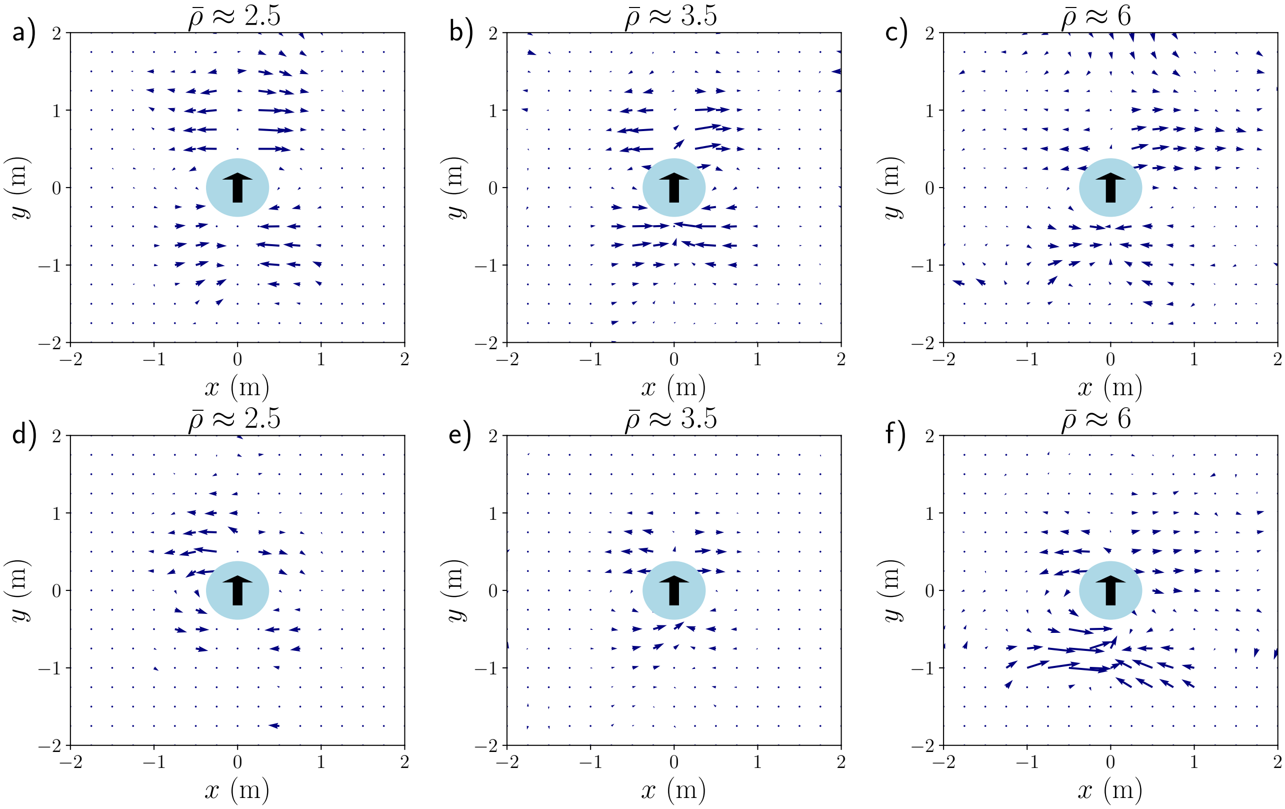

Figure S1. Mean velocity fields (based on the displacements over a time window $\delta t = 0.$ s) around the cylinder in crowds of participants facing the intruder (top row) and randomly oriented ones (bottom row) at **(a-d)** moderate density ($\bar{\rho} \approx 2.5\,\mathrm{ped/m^2}$), **(b-e)** fairly high density ($\bar{\rho} \approx 3.5\,\mathrm{ped/m^2}$), and **(c-f)** very high density ($\bar{\rho} \approx 6\,\mathrm{ped/m^2}$). The velocity arrows on the left, middle, and right panels are magnified by a factor 1, 2, and 4, respectively.

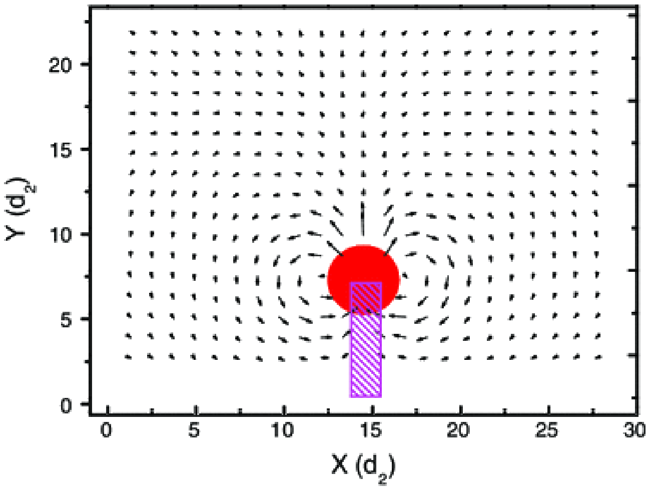

Figure S2. Mean velocity field around a circular intruder in a two-dimensional granular medium. Adapted from Ref.[1], with permission.

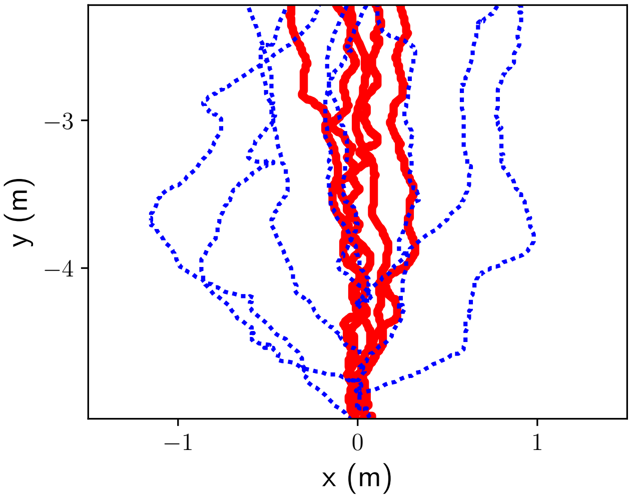

Figure S3. Traces of a few typical trajectories of the cylindrical intruder (thick red lines) and of crossing pedestrians (dotted blue lines) through a fairly dense crow ($\rho \approx 3.5\,\mathrm{ped}/\mathrm{m}^2$) facing the intruder. The trajectories have been shifted horizontally to start from $x = 0$.

References

1. Kolb, E., Cixous, P. & Charmet, J. Flow fields around an intruder immersed in a 2d dense granular layer. *Granul. Matter* **16**, 223–233 (2014).
